# Contemporary Applications of Platelet-Rich Fibrin in Mandibular Surgery With Implications for Mandibular Fracture Management

**DOI:** 10.7759/cureus.113974

**Published:** 2026-08-04

**Authors:** Nima Varghese, Yash D Shah, Sabitha Sri Imandi, Shweta Chhaya, Devang Patel, Girish S Shelke

**Affiliations:** 1 Department of Dentistry, Goregaon Dental Centre, Mumbai, IND; 2 Department of Oral Biology, School of Dental Medicine, University at Buffalo, New York City, USA; 3 Department of Orthodontics and Dentofacial Orthopedics, Yerala Medical Trust (YMT) Dental College and Hospital, Dr. G.D. Pol Foundation, Navi Mumbai, IND; 4 Department of Dentistry, Karnavati School of Dentistry, Gandhinagar, IND; 5 Department of Dentistry, Choctaw Nation of Oklahoma, Indian Health Service, Durant, USA

**Keywords:** bone regeneration, fracture healing, mandibular fractures, oral and maxillofacial surgeries, platelet-rich fibrin

## Abstract

Mandibular surgery encompasses a wide range of procedures, including the management of traumatic fractures and elective osteotomies, where optimal bone healing and soft-tissue regeneration are essential for successful clinical outcomes. Platelet-rich fibrin (PRF), a second-generation autologous platelet concentrate, has gained increasing attention because of its ability to provide a fibrin scaffold enriched with platelets, leukocytes, cytokines, and growth factors that promote angiogenesis, osteogenesis, and soft-tissue regeneration. This narrative review summarizes the current evidence regarding the biological basis, clinical applications, and therapeutic role of PRF in mandibular surgery, with particular emphasis on its implications for mandibular fracture management. A structured literature search of major electronic databases was performed to identify relevant studies evaluating PRF in mandibular surgical procedures, including traumatic mandibular fractures and elective mandibular osteotomies. The available evidence suggests that PRF may enhance radiographic bone healing, improve soft-tissue repair, reduce postoperative inflammation, and facilitate neurosensory recovery when used as an adjunct to conventional surgical management. However, the evidence specific to traumatic mandibular fractures remains limited, with much of the available clinical data derived from elective mandibular surgical procedures, including bilateral sagittal split osteotomy and related osteotomies. Furthermore, considerable heterogeneity exists among published studies with respect to PRF preparation protocols, application techniques, surgical indications, and outcome measures, limiting direct comparisons and definitive clinical recommendations. Current evidence suggests that PRF is a safe, relatively low-cost, and biologically promising adjunct in mandibular surgery; however, fracture-specific clinical evidence remains limited. Further well-designed multicenter randomized clinical trials employing standardized PRF preparation protocols and long-term follow-up, particularly in patients with traumatic mandibular fractures, are required to establish evidence-based recommendations for routine clinical use.

## Introduction and background

Mandibular fractures are among the most common maxillofacial injuries encountered in clinical practice and account for a substantial proportion of facial trauma worldwide. These fractures frequently result from road traffic accidents, interpersonal violence, falls, and sports-related injuries, and can significantly impair mastication, speech, occlusion, facial esthetics, and overall quality of life [1]. The primary objectives of mandibular fracture management are to restore anatomical continuity, achieve stable occlusion, preserve mandibular function, and minimize postoperative complications [2]. Over the past three decades, remarkable advances in diagnostic imaging, surgical techniques, and fixation systems have transformed the management of mandibular fractures from prolonged maxillomandibular fixation to rigid and semi-rigid internal fixation, enabling earlier functional rehabilitation and improved clinical outcomes [3]. Nevertheless, complications such as delayed bone healing, infection, wound dehiscence, neurosensory disturbances, and non-union continue to challenge clinicians, particularly in complex or comminuted fractures [4].

In addition to biomechanical innovations, increasing attention has been directed toward biological approaches that enhance bone and soft-tissue healing following mandibular surgical procedures. Platelet-rich fibrin (PRF), a second-generation autologous platelet concentrate, has emerged as a promising regenerative biomaterial owing to its ease of preparation, relatively low cost, autologous nature, and favorable biological properties [5]. PRF provides a three-dimensional fibrin scaffold enriched with platelets, leukocytes, cytokines, and growth factors, including platelet-derived growth factor, transforming growth factor-β, and vascular endothelial growth factor, which collectively promote angiogenesis, osteogenesis, soft-tissue regeneration, and modulation of the inflammatory response [6]. These biological characteristics have led to its widespread application across various oral and maxillofacial surgical procedures, including implant dentistry, periodontal regeneration, sinus augmentation, alveolar ridge preservation, orthognathic surgery, and other mandibular surgical interventions. More recently, PRF has also been investigated as an adjunct in the management of traumatic mandibular fractures to enhance bone healing, improve soft-tissue repair, and reduce postoperative complications [7].

Although several experimental and clinical studies suggest that PRF may enhance bone regeneration, reduce postoperative infection, and potentially improve neurosensory recovery following mandibular fractures, the available evidence remains heterogeneous because of variations in PRF preparation protocols, application techniques, study designs, and outcome measures [8,9]. Consequently, despite encouraging preliminary findings, insufficient high-quality clinical evidence and the absence of standardized PRF protocols continue to limit its routine incorporation into mandibular fracture management. This narrative review summarizes the current concepts in mandibular fracture management, discusses the biological rationale and clinical applications of PRF, critically evaluates the available evidence supporting its use in mandibular fractures, identifies existing knowledge gaps, and highlights future directions for research and clinical practice.

## Review

A structured literature search was conducted to identify relevant publications evaluating the applications of platelet-rich fibrin (PRF) in mandibular surgery, including traumatic mandibular fractures and elective mandibular surgical procedures. Electronic databases, including PubMed/MEDLINE, Scopus, Web of Science, and Google Scholar, were searched for articles published up to May 2026. The reference lists of selected articles were manually screened to identify additional relevant publications. The search strategy combined Medical Subject Headings (MeSH) and free-text keywords using Boolean operators. The principal search terms were as follows: "Mandibular Fractures"[MeSH], "Platelet-Rich Fibrin", "Platelet Rich Fibrin", "PRF", "Orthognathic Surgery", "Bilateral Sagittal Split Osteotomy", "Sagittal Split Ramus Osteotomy", "Oral Surgical Procedures", "Oral and Maxillofacial Surgery", "Maxillofacial Injuries", "Bone Regeneration", "Fracture Healing", and "Open Reduction Internal Fixation". These terms were combined using the Boolean operators AND and OR to maximize the retrieval of relevant studies.

**Figure 1:**
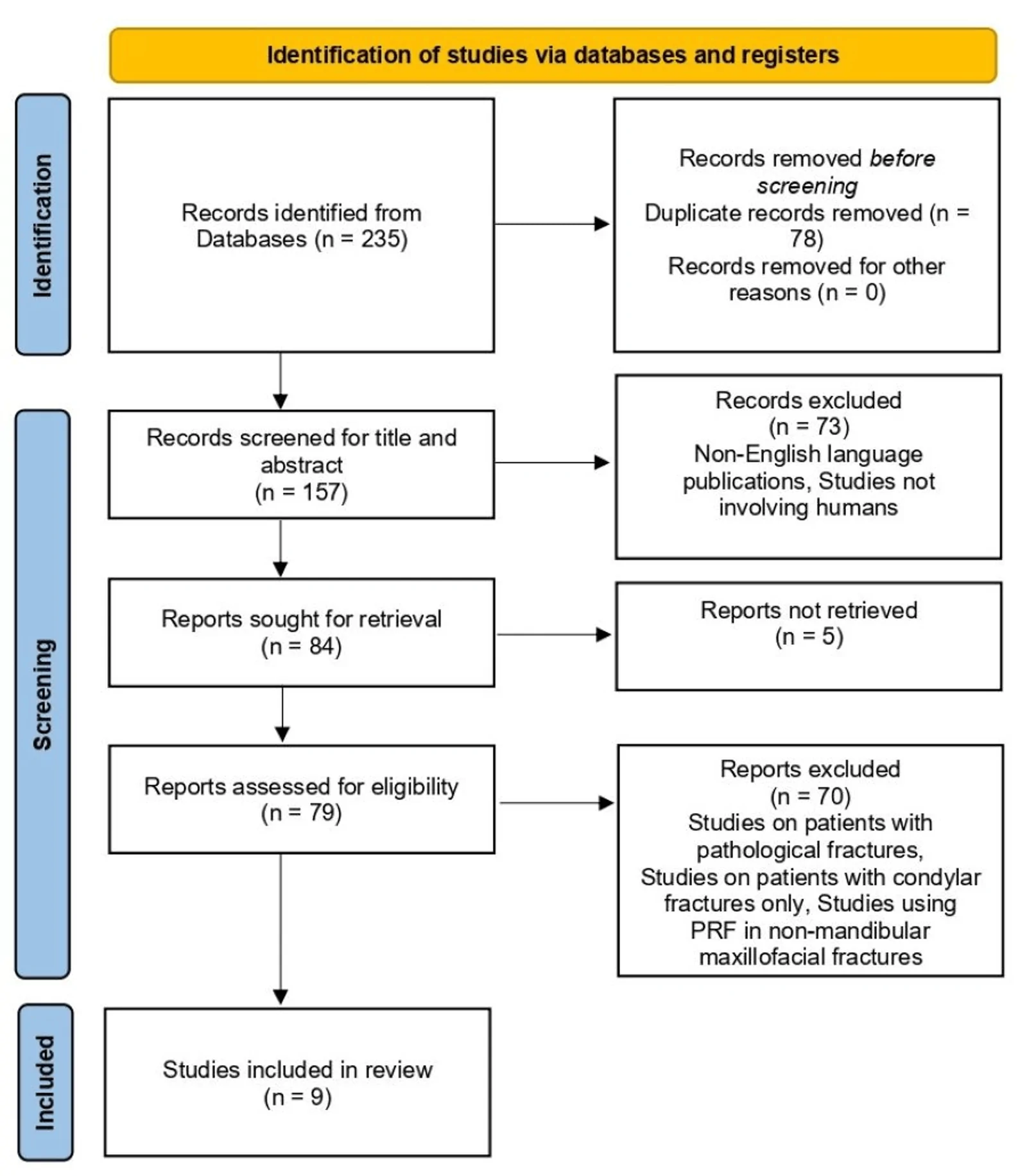
PRISMA 2020 flow diagram illustrating the study selection process. PRF: platelet-rich fibrin; PRISMA: Preferred Reporting Items for Systematic Reviews and Meta-Analyses

Original clinical studies, randomized controlled trials, prospective and retrospective cohort studies, comparative studies, systematic reviews, meta-analyses, narrative reviews, and relevant experimental studies published in English were included. Articles focusing on mandibular surgical procedures, including traumatic mandibular fracture management, fracture fixation techniques, orthognathic surgery, regenerative approaches, PRF applications, bone healing, infection control, and neurosensory recovery were included. Editorials, conference abstracts, letters to the editor, duplicate publications, studies lacking sufficient methodological details, and articles unrelated to mandibular fractures or platelet-rich fibrin were excluded. The titles and abstracts of all retrieved records were independently screened for relevance, followed by a full-text assessment of potentially eligible articles. Study screening was performed independently by two reviewers. Any discrepancies regarding study eligibility were resolved through discussion and consensus between the reviewers; therefore, involvement of a third reviewer was not required. The available evidence was subsequently synthesized narratively, with emphasis on the evolution of mandibular fracture management, the biological basis of PRF, current clinical applications, regenerative outcomes, limitations of existing evidence, and future research directions (Figure 1).

Research trends

**Figure 2:**
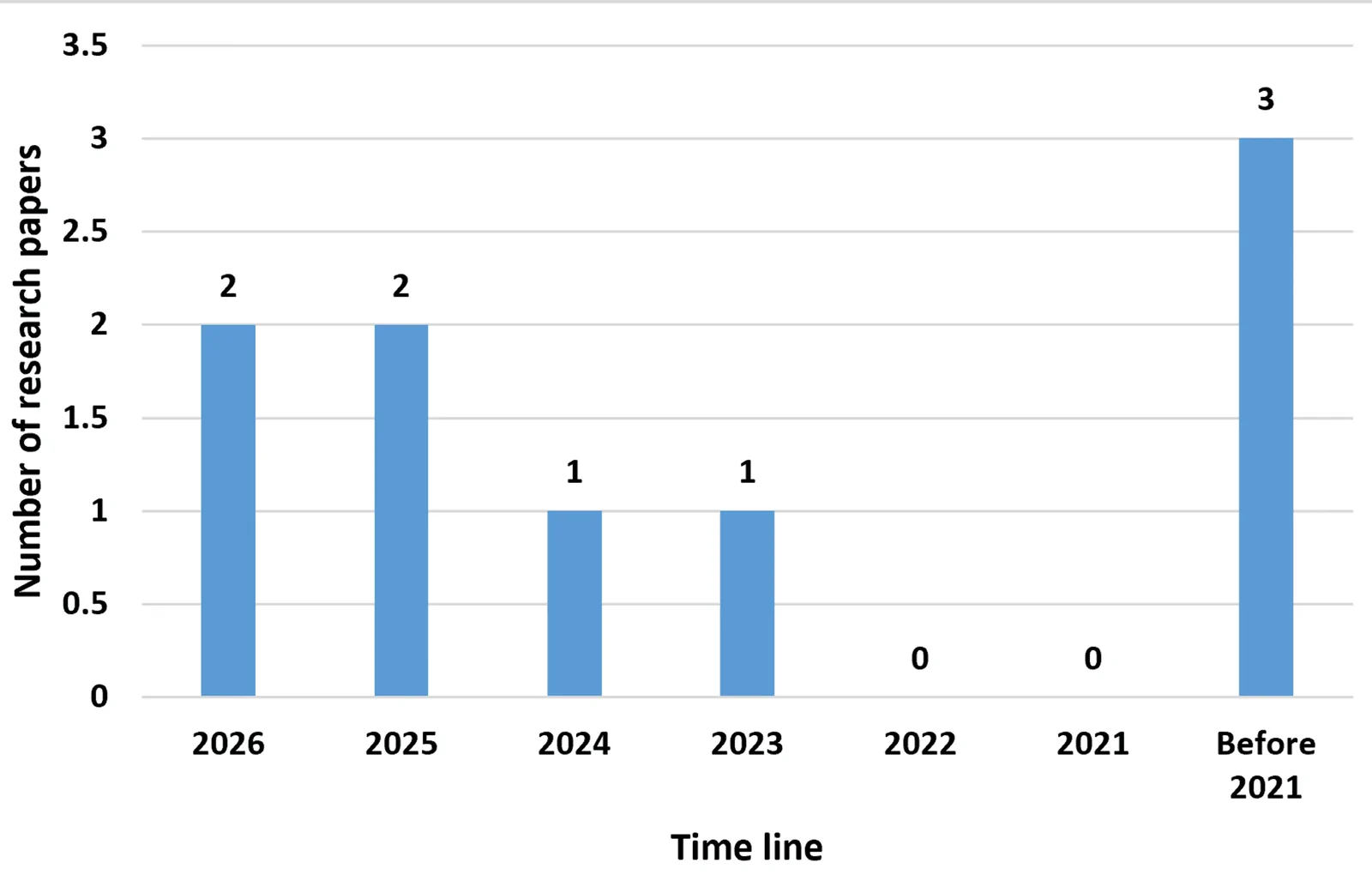
Year-wise distribution of the nine eligible studies included in this narrative review on the role of platelet-rich fibrin in mandibular fracture and surgical management.

Figure 2 illustrates the temporal distribution of publications that investigated the role of PRF in mandibular fracture management. The available literature demonstrates that this is a relatively emerging area of research with only a limited number of studies published over the past decade. Although the number of publications remains limited, an increase in studies published since 2023 suggests growing interest in evaluating PRF as an adjunct in mandibular fracture and surgical management. The limited number of publications also highlights the need for additional high-quality clinical investigations to establish standardized treatment protocols and strengthen the current evidence base.

Evolution of mandibular fracture management

Mandibular fractures are among the most frequently encountered injuries in oral and maxillofacial trauma, accounting for a substantial proportion of facial fractures worldwide [1]. Their management has evolved remarkably over the past several decades, driven by advances in imaging modalities, fixation systems, biomaterials, and a better understanding of mandibular biomechanics. The primary goals of treatment are anatomical reduction of fracture fragments, restoration of preinjury occlusion, achievement of stable fixation, early functional rehabilitation, and prevention of postoperative complications, such as infection, malunion, non-union, and neurosensory deficits [10].

Historically, mandibular fractures have been managed predominantly by prolonged maxillomandibular fixation (MMF), which relies on immobilization to facilitate fracture healing [11,12]. Although effective in maintaining occlusion, extended immobilization is associated with several disadvantages, including compromised oral hygiene, nutritional deficiencies, airway concerns, temporomandibular joint stiffness, muscle atrophy, and delayed return to normal function [13]. These limitations have prompted the development of more stable fixation techniques that permit early mobilization while maintaining fracture stability.

Open reduction and internal fixation (ORIF) has revolutionized mandibular fracture management. The widespread adoption of titanium miniplates and screws, based on Champy's principles of functional osteosynthesis, enables rigid or semi-rigid fixation with predictable clinical outcomes [14]. Modern fixation strategies are now tailored according to the fracture location, displacement, biomechanical loading, and patient-specific factors. Load-sharing fixation using miniplates is commonly employed for favorable fractures, whereas load-bearing reconstruction plates are preferred for comminuted fractures, segmental defects, atrophic mandibles, and cases involving significant bone loss [2]. Recent advances, including locking plate systems, three-dimensional plates, patient-specific implants, and computer-assisted surgical planning, have further improved fixation accuracy, while reducing operative morbidity [15].

Despite these biomechanical advances, postoperative complications remain a major clinical concern. Delayed bone healing, infection, hardware failure, wound dehiscence, and inferior alveolar nerve dysfunction continue to affect treatment outcomes, particularly in patients with high-energy trauma, systemic comorbidities, or compromised vascularity [16]. Consequently, contemporary research has shifted beyond mechanical stabilization alone to biological enhancement of fracture healing. This transition has stimulated considerable interest in regenerative approaches capable of accelerating osseous regeneration, while simultaneously improving soft-tissue healing and reducing postoperative complications. Among these biological adjuncts, platelet-rich fibrin has emerged as one of the most promising autologous regenerative biomaterials for oral and maxillofacial surgery [8,9].

Biological basis of platelet-rich fibrin

PRF is a second-generation autologous platelet concentrate developed to enhance tissue regeneration through sustained release of biologically active growth factors. Unlike platelet-rich plasma, PRF is prepared without anticoagulants or exogenous thrombin, allowing the natural polymerization of fibrin during centrifugation [5,6]. This process produces a dense three-dimensional fibrin matrix that entraps platelets, leukocytes, cytokines, circulating stem cells, and numerous growth factors, thereby functioning as both a biological scaffold and reservoir for regenerative mediators [5].

The regenerative potential of PRF is primarily attributed to its gradual release of platelet-derived growth factor (PDGF), transforming growth factor-β (TGF-β), vascular endothelial growth factor (VEGF), insulin-like growth factor (IGF), epidermal growth factor (EGF), and other bioactive molecules over several days [17]. These mediators regulate multiple stages of wound healing, including cellular migration, angiogenesis, extracellular matrix synthesis, collagen deposition, osteoblastic differentiation, and mineralized tissue formation. Simultaneously, the leukocyte-rich fibrin network contributes to immunomodulation, antimicrobial activity, and regulation of the inflammatory response, thereby creating a favorable microenvironment for fracture repair [18].

Experimental studies have consistently demonstrated that PRF enhances osteoblast proliferation, increases alkaline phosphatase activity, stimulates mineralized nodule formation, and promotes angiogenesis during early bone healing. Furthermore, the fibrin matrix serves as a natural scaffold that facilitates the migration of mesenchymal stem cells and endothelial cells into healing tissue, thereby accelerating both hard and soft-tissue regeneration [5]. These biological properties have led to widespread clinical applications of PRF in periodontal regeneration, implant dentistry, sinus floor augmentation, alveolar ridge preservation, management of medication-related osteonecrosis of the jaw, endodontic regeneration, and oral soft-tissue reconstruction [7,9,19,20].

In the context of mandibular fractures, PRF has attracted increasing attention as a biologically active adjunct capable of complementing conventional osteosynthesis rather than replacing mechanical fixation [9]. The rationale for its use is based on its ability to accelerate bone formation, improve vascularization of the fracture site, reduce postoperative inflammation, decrease the incidence of surgical site infection, and potentially enhance regeneration of injured peripheral nerves [21]. Although encouraging clinical evidence has emerged over the past decade, considerable variability exists in PRF preparation protocols, centrifugation parameters, formulation types, application techniques, and outcome assessment methods. Consequently, although the biological rationale supporting PRF is well established, further high-quality clinical evidence is required before standardized recommendations for its routine use in mandibular fracture management can be established.

Clinical applications of platelet-rich fibrin in oral and maxillofacial surgery

The regenerative properties of PRF have resulted in its widespread application in various disciplines of oral and maxillofacial surgery [9]. PRF has become an attractive adjunct in procedures requiring enhanced soft-tissue healing and bone regeneration owing to its autologous origin, ease of preparation, minimal cost, and favorable safety profile. Unlike synthetic biomaterials or recombinant growth factors, PRF utilizes the patient's biological components, thereby minimizing the risk of immunological reactions, disease transmission, and foreign body responses [6].

Among the most extensively investigated applications is alveolar ridge preservation following tooth extraction [20,22]. PRF accelerates soft-tissue closure, reduces postoperative pain, preserves alveolar bone dimensions, and improves the quality of newly formed bone [23]. Similarly, in implant dentistry, PRF has been used around implant sites to enhance osseointegration, promote peri-implant soft-tissue healing, and improve early implant stability [7]. In maxillary sinus augmentation procedures, PRF serves both as a biologically active scaffold and a carrier for particulate bone grafts, promoting angiogenesis and accelerating graft maturation [24].

PRF has also demonstrated promising outcomes in periodontal regenerative procedures, including the treatment of intrabony defects, gingival recession, and furcation involvement, where it enhances periodontal ligament regeneration and connective tissue attachment [25]. Furthermore, successful clinical applications have been reported in the management of medication-related osteonecrosis of the jaw, oroantral communication, cystic defects, and reconstructive surgeries involving bone grafts [26]. Collectively, these applications demonstrate that PRF consistently enhances wound healing by stimulating angiogenesis, collagen synthesis, cellular proliferation, and the controlled release of growth factors. The accumulated clinical experience obtained from these procedures has provided the biological foundation for extending PRF applications to maxillofacial trauma surgery, particularly in the management of mandibular fractures, where rapid bone healing and prevention of postoperative complications are of paramount importance [8,9].

Platelet-rich fibrin in mandibular fracture healing

Nine studies included in the review are shown in Table 1 [8,9,27-33]. The incorporation of PRF into mandibular fracture management represents an important advancement in biologically assisted osteosynthesis. Although stable mechanical fixation remains the cornerstone of successful fracture treatment, biological enhancement has emerged as a complementary strategy aimed at optimizing the local healing environment. PRF is typically applied directly along the fracture line or beneath the periosteum during open reduction and internal fixation, where it acts as a natural fibrin scaffold capable of sustaining the release of growth factors throughout the early stages of fracture repair [9].

**Table 1:** List of included studies in review. PRF: platelet-rich fibrin; T-PRF: titanium-prepared platelet-rich fibrin

S. no.	Studies	Year	PRF	Fracture/surgery
1	Sakarinpanichakul and Burapholkul [9]	2026	Platelet-rich fibrin	Mandibular fracture
2	Elyamany et al. [27]	2026	Photobiomodulation-enhanced T-PRF	Mandibular fracture
3	Zhu et al. [28]	2025	Advanced platelet-rich fibrin	Mandibular bilateral sagittal split osteotomy
4	Micko et al. [29]	2025	Injectable platelet-rich fibrin	Mandibular bilateral sagittal split osteotomy
5	Tabrizi et al. [8]	2024	Platelet-rich fibrin	Mandibular fracture
6	Behnia et al. [30]	2023	Leukocyte- and platelet-rich fibrin	Mandibular genioplasty
7	Esenlik et al. [31]	2019	Platelet-rich fibrin	Mandibular bilateral segmental osteotomy
8	Al-Khawlani et al. [32]	2014	Platelet-rich fibrin gel	Mandibular fracture
9	Al-Khawlani et al. [33]	2014	Platelet-rich fibrin	Mandibular fracture

The biological rationale for PRF application is its ability to accelerate each phase of bone healing. Immediately following fracture stabilization, the fibrin matrix supports platelet activation and leukocyte migration, initiating a controlled inflammatory response that is essential for tissue repair. Subsequently, the sustained release of platelet-derived growth factor, transforming growth factor-β, vascular endothelial growth factor, and insulin-like growth factor promotes angiogenesis, recruitment of osteoprogenitor cells, osteoblastic differentiation, and extracellular matrix deposition. Improved neovascularization enhances oxygen and nutrient delivery to the fracture site, facilitating earlier callus maturation and mineralization [34].

Experimental and clinical investigations have demonstrated favorable outcomes following PRF application for mandibular fracture fixation. Studies evaluating PRF in various oral and maxillofacial applications have demonstrated improved radiographic bone regeneration, enhanced soft-tissue healing, and favorable postoperative outcomes. Evidence from elective mandibular surgical procedures, such as bilateral sagittal split osteotomy, further supports the regenerative potential of PRF; however, these findings represent indirect evidence and should not be interpreted as fracture-specific outcomes [28,29,31]. The leukocyte-rich fibrin network further contributes to local antimicrobial defense while modulating excessive inflammatory responses that may otherwise delay bone regeneration. Emerging evidence also suggests a potential role for PRF in promoting regeneration of the inferior alveolar nerve following traumatic injury, although current evidence remains limited [8,9].

Despite these encouraging findings, the available literature remains heterogeneous with respect to centrifugation protocols, PRF formulations, application techniques, fracture characteristics, fixation methods, and outcome assessments [35]. Consequently, although PRF appears to be a promising adjunctive biomaterial capable of enhancing biological healing without altering conventional surgical principles, standardized preparation protocols and well-designed multicenter clinical trials are required before routine incorporation into mandibular fracture management is universally recommended.

Clinical evidence supporting platelet-rich fibrin in mandibular fractures

Over the past decade, an increasing number of clinical investigations have evaluated PRF as an adjunct to conventional open reduction and internal fixation for mandibular fractures [7,9]. Although the available evidence remains limited, the collective findings suggest that PRF may positively influence several aspects of fracture healing, particularly early bone regeneration and postoperative tissue repair. Supporting the regenerative potential of platelet concentrates in mandibular surgery, Zhu et al. conducted a double-blind, split-mouth randomized clinical trial and demonstrated that advanced PRF (A-PRF) significantly reduced postoperative pain, facial swelling, drainage, and inferior alveolar nerve sensory disturbances following sagittal split ramus osteotomy, while also improving bone mineral density within the osteotomy gap [28]. These findings suggest that PRF not only enhances soft-tissue healing but also promotes early bone regeneration, reinforcing its potential as an adjunctive biomaterial in the management of mandibular fractures and other maxillofacial surgical procedures.

Further supporting the regenerative role of platelet concentrates, Micko et al. demonstrated that injectable PRF (I-PRF) significantly increased bone volume within the bilateral sagittal split osteotomy gap on cone-beam computed tomography compared with conventional surgery [29]. The authors attributed these findings to the sustained release of growth factors, osteogenic potential, and antibacterial properties of I-PRF, highlighting its value as a simple autologous adjunct for enhancing bone regeneration and minimizing postoperative mandibular contour defects, with potential implications for mandibular fracture management. Behnia et al. demonstrated that leukocyte- and platelet-rich fibrin (L-PRF) significantly accelerated early neurosensory recovery after genioplasty, improving two-point discrimination and reducing patient-reported sensory disturbances during the first four postoperative months [30]. These findings support the role of PRF in promoting peripheral nerve healing following mandibular surgery.

One of the most consistently reported benefits of PRF is the enhancement of radiographic bone healing [29,36]. Clinical studies in oral surgical procedures, including endodontic microsurgery, have demonstrated significantly greater radiographic bone density and enhanced early bone healing following PRF application, supporting its regenerative potential. These findings, however, represent indirect evidence with respect to traumatic mandibular fracture management [36]. These findings have been attributed to the sustained release of osteogenic growth factors that stimulate osteoblast proliferation, collagen synthesis, angiogenesis, and mineralization. Improved vascularization within the fracture gap further facilitates nutrient delivery and cellular migration, accelerating the normal cascade of bone repair [37].

In addition to promoting bone regeneration, PRF reduces postoperative complications. Several investigators have reported lower rates of surgical site infection, improved soft-tissue healing, and reduced wound dehiscence following PRF application [37]. The regenerative potential of platelet-rich fibrin is attributed in part to its sustained release of growth factors, including vascular endothelial growth factor (VEGF), which promotes angiogenesis, tissue repair, and bone regeneration during the healing process [38]. The leukocyte-rich fibrin matrix provides antimicrobial activity while simultaneously modulating the inflammatory response, thereby creating a favorable environment for tissue regeneration [5]. Although some oral surgical studies have reported reduced postoperative pain following the use of autologous platelet concentrates, findings remain inconsistent, with several investigations demonstrating no statistically significant benefit compared with conventional treatment. These observations represent indirect evidence and should not be extrapolated directly to traumatic mandibular fracture management [39]. Another emerging area of interest is the influence of PRF on neurosensory recovery after mandibular fractures involving the inferior alveolar nerve [8,9]. Preliminary clinical evidence suggests that PRF may enhance peripheral nerve regeneration by providing neurotrophic growth factors and reducing perineural fibrosis [40]. However, the available studies are few, involve relatively small patient populations, and use different methods for neurosensory assessment. Consequently, the current evidence should be interpreted cautiously until it is validated by larger randomized controlled trials.

A recent systematic review and meta-analysis by Sivaramakrishnan et al. included 14 studies and demonstrated that the adjunctive use of PRF significantly improved bone mineral density at both the three- and six-month follow-ups in mandibular fractures [21]. This review also reported favorable clinical outcomes, including reduced inflammation, enhanced soft-tissue healing, greater pain relief, and improved jaw mobility, although the authors emphasized the need for further high-quality clinical trials to strengthen the evidence. Esenlik et al. reported that PRF augmentation during segmental corticotomy-assisted orthodontic treatment enhanced alveolar bone support, promoted vertical bone gain, and maintained periodontal health, demonstrating the regenerative capacity of PRF in mandibular bone remodeling and healing [31].

Al-Khawlani et al. demonstrated that local application of PRF at the mandibular fracture site accelerated bone healing without any treatment-related adverse effects, supporting PRF as a safe and effective adjunct to open reduction and internal fixation of mandibular fractures [32]. In a prospective comparative study, Al-Khawlani et al. found that both PRP and PRF enhanced bone healing in mandibular fractures; however, PRF demonstrated superior bone formation, suggesting that it may be the more effective autologous platelet concentrate for promoting fracture regeneration [33]. Overall, the available clinical literature supports the use of PRF as a promising biological adjunct to complement conventional osteosynthesis. Nevertheless, existing evidence is insufficient to establish standardized clinical protocols, emphasizing the need for further high-quality prospective studies.

Challenges and limitations

Despite encouraging clinical outcomes, several challenges continue to limit the widespread adoption of PRF for mandibular fracture management. The most significant limitation was the absence of standardized preparation protocols. Variations in blood collection techniques, centrifugation speed, centrifugation duration, rotor characteristics, tube composition, and processing time substantially influence cellular composition, fibrin architecture, and concentration of released growth factors. Consequently, PRF prepared using different protocols may exhibit different biological properties, making direct comparisons among published studies difficult [35]. Although evidence from other autologous platelet concentrates, such as platelet-rich plasma, suggests a potential role in peripheral nerve repair through the delivery of bioactive growth factors, these findings cannot be directly extrapolated to platelet-rich fibrin because of differences in their preparation methods, fibrin architecture, cellular composition, and growth-factor release kinetics [41].

Another important limitation is the considerable heterogeneity of existing clinical investigations. Studies differ with respect to fracture location, severity, fixation techniques, timing of PRF application, follow-up duration, and outcome assessment methods. Furthermore, the sample sizes remain relatively small, and many studies are single-center investigations with limited statistical power. Such methodological variability restricts the generalizability of the current findings and contributes to inconsistent conclusions regarding their clinical effectiveness. Patient-related variables also influence treatment outcomes. Age, smoking status, systemic diseases such as diabetes mellitus, nutritional status, and the severity of trauma can independently affect bone healing and may confound the regenerative effects attributed to PRF [42]. Additionally, differences in postoperative care, antibiotic protocols, and rehabilitation further complicate the interpretation of treatment outcomes.

Although PRF is inexpensive, autologous, and associated with an excellent safety profile, preparation requires blood collection and immediate centrifugation before surgery, which may increase operative time and require additional equipment and trained personnel [35]. These practical considerations may limit the routine implementation in emergency trauma settings with limited resources. Therefore, future investigations should prioritize standardized PRF preparation protocols, uniform outcome measures, adequately powered multicenter randomized controlled trials, and long-term follow-up to establish evidence-based recommendations for routine incorporation into mandibular fracture management.

Future perspectives and research directions

The application of platelet-rich fibrin in mandibular fracture management represents an evolving area of regenerative maxillofacial surgery, with considerable potential for future development. Although current evidence suggests that PRF may enhance bone regeneration and improve selected postoperative outcomes, its widespread clinical adoption is limited by a lack of standardized preparation protocols and robust clinical evidence. Therefore, future investigations should focus on establishing consensus guidelines regarding blood collection techniques, centrifugation parameters, tube composition, and methods of PRF application to ensure reproducibility and facilitate meaningful comparisons among studies.

Emerging modifications of conventional PRF, including advanced platelet-rich fibrin (A-PRF), I-PRF, L-PRF, and titanium-prepared platelet-rich fibrin (T-PRF), have demonstrated promising biological characteristics that may further enhance tissue regeneration [43]. Comparative clinical studies are required to determine whether these newer formulations provide superior regenerative outcomes compared to conventional PRF in mandibular fracture healing. Furthermore, combining PRF with other regenerative strategies such as bone graft substitutes, mesenchymal stem cells, recombinant growth factors, low-level laser therapy, and three-dimensional printed biomaterial scaffolds may offer synergistic effects that accelerate fracture repair and improve functional recovery. Elyamany et al. reported improved osseous regeneration with increased bone density at 12 weeks and greater pain reduction compared with T-PRF alone [27].

Future research should also prioritize adequately powered multicenter randomized controlled trials with standardized outcome measures, longer follow-up periods, and objective radiographic assessments using cone-beam computed tomography or computed tomography-based bone density analysis. Patient-reported outcome measures, neurosensory recovery, quality of life, functional rehabilitation, and cost-effectiveness should be incorporated as clinically meaningful end points. The integration of artificial intelligence and digital image analysis may further improve the quantitative assessment of fracture healing and facilitate individualized treatment planning. Collectively, these advances have the potential to establish evidence-based recommendations and define the precise role of PRF in contemporary mandibular fracture management.

Clinical implications

Current evidence suggests that PRF is a promising biological adjunct in mandibular surgery because of its potential to enhance bone regeneration and soft-tissue healing. However, the available clinical evidence specific to traumatic mandibular fractures remains limited, and a substantial proportion of the published literature is derived from elective mandibular surgical procedures. Therefore, PRF should be considered an adjunct to established surgical principles rather than a substitute for stable fracture fixation or meticulous surgical technique. Although the biological properties of PRF suggest that it may be beneficial in clinical situations where enhanced tissue regeneration is desirable, there is currently insufficient comparative evidence to identify specific patient populations that derive greater benefit from its use. Consequently, the selection of patients for PRF application should be based on individual clinical judgment until higher-quality evidence becomes available. Future well-designed, adequately powered randomized controlled trials should evaluate the effectiveness of PRF in different mandibular fracture patterns and patient populations, including individuals with compromised healing potential, to determine whether particular clinical subgroups derive additional benefit from biological augmentation.

## Conclusions

Platelet-rich fibrin has emerged as a promising biological adjunct in mandibular surgery because of its ability to enhance bone regeneration, promote soft-tissue healing, and potentially reduce postoperative complications. Evidence from both traumatic mandibular fracture studies and elective mandibular surgical procedures supports its regenerative potential; however, the evidence specific to traumatic mandibular fractures remains limited. Furthermore, variations in PRF preparation protocols, application techniques, and study methodologies, together with the limited availability of high-quality clinical trials, preclude routine universal use. Accordingly, PRF should be considered a valuable adjunct rather than a replacement for established surgical principles and stable fixation where indicated. Future well-designed, standardized, multicenter clinical trials, particularly in patients with traumatic mandibular fractures, are essential to establish evidence-based protocols and define the precise role of PRF in mandibular surgery and maxillofacial trauma care.
